# A Relative Measurement of Oxidative Stress in NAFLD Through Cyclic Voltammetry Method for Clinical Translation

**DOI:** 10.1155/grp/9948444

**Published:** 2025-04-16

**Authors:** Dixa Sharma, Bhalendu S. Vaishnav, Nupur Pandya, Pratik Pataniya, C. K. Sumesh, Palash Mandal

**Affiliations:** ^1^Department of Biological Sciences, P D Patel Institute of Applied Sciences, Charotar University of Science and Technology, Anand, Gujarat, India; ^2^H M Patel Centre for Medical Care and Education, Charutar Arogya Mandal, Karamsad, India; ^3^Department of Physical Sciences, P D Patel Institute of Applied Sciences, Charotar University of Science and Technology, Anand, Gujarat, India

**Keywords:** cyclic voltammetry, NAFLD, oxidative stress, personalized therapeutics, risk assessment

## Abstract

A potential contributing factor in the development of various metabolic diseases such as nonalcoholic fatty liver disease (NAFLD) could be oxidative stress and the production of reactive oxygen radicals. A high level of lipid peroxidation, including oxidative stress, can cause irreversible effects. We investigated the consequences of NAFLD on the reducing power of the liver in patients through plasma antioxidant capacity using screen-printed electrodes (SPEs). The study includes a total of 67 patient's population with steatosis (*n* = 29) and steatohepatitis (*n* = 38). Anodic current intensity (*la*), anodic wave area (*S*), and the biological sample oxidation potentials can be determined via cyclic voltammetry (CV) analysis. The enzyme glutathione peroxidase (GPx) and products of oxidative damage such as malondialdehyde (MDA), advanced glycation-end product (AGE), total status of oxidants (TOS), nitric oxide (NO), and cytokines analysis (qRT-PCR) of key mediators such as PNPLA3 in lipid metabolism, TIMP1 in fibrosis, and proinflammatory cytokines like NF-*κ*B, TNF-*α*, and IL-6, which are crucial for understanding NAFLD progression were recorded to further validate the CV obtained results along with and morphological changes through scanning electron microscope (SEM). The developed method measured oxidative stress with an error of less than 1.3% in human plasma samples, wherein the steatohepatitis caused a spike modification in the anodic current AC_520_ and AC_972_ (*p* < 0.01) compared to healthy humans. The presented electroanalytical methodology could be widely used for easy and rapid subjects' disease status detection. In addition to monitoring the response of subjects to treatment and providing nutritional supplements, these results may also be used for screening specific populations.

## 1. Introduction

NAFLD is a multifactorial and complex disease. There is widespread mortality and morbidity associated with this around the world. Approximately 25%–33% of adults living in developed countries suffer from NAFLD [1]. Fatty liver disease is expected to become more prevalent in the future [2]. Hepatic steatosis is a trait associated with NAFLD ranging from mild to modest effects governed by multiple variants [3]. A well-known determinant of NAFLD is the low levels of antioxidants. The disease diagnosis is based on assessment such as glutathione peroxidase (GPx), alanine transaminase (ALT), aspartate aminotransferase (AST), computed tomography (CT), ultrasound sonography test (USG), and biopsy to eliminate the other causes of liver damage [4]. It aimed to investigate whether antioxidants are associated with specific histological USG patterns, activation of the HpSC niche, and levels of oxidative stress in serum when measuring the liver's reductive capacity. Furthermore, the roles of key mediators like Patatin-like phospholipase domain-containing protein 3 (PNPLA3) in lipid metabolism, tissue inhibitor of metalloproteinases 1 (TIMP1) in fibrosis, nitric oxide (NO) in redox signaling, and proinflammatory cytokines such as TNF-*α* and IL-6, and advanced oxidation protein products (AOPPs) which are implicated in NAFLD pathogenesis, has been assessed. NO exhibits a complex and context-dependent role in NAFLD, capable of both promoting and inhibiting the formation of AGEs. Furthermore, NO influences lipid peroxidation through diverse mechanisms and contributes to both pro-oxidant and antioxidant effects, reflecting its multifaceted impact on the disease's pathophysiology. Among the techniques used to detect antioxidants are paramagnetic [5], colorimetric [6], and electrochemical sensing [7]. Aside from having high sensitivity and selectivity, electrochemical sensors are the most cost-effective technique for oxidative stress detection [8].

One of the fundamental characteristics of NAFLD progression and its related systemic conditions is oxidative stress, but in a clinical setting, this state is not measured routinely. Reactive oxygen species (ROS) are produced when mitochondrial dysfunction occurs, neutrophils become activated, and transition metals are released during primary injury [9]. ROS induces nuclear factor kappa light chain enhancer of activated B cells (NF-*κ*B) activation, disrupting redox signaling, and leading to apoptosis (via caspase 3), plasma membrane rupture (ferroptosis), and further ROS generation, all of which contribute to NAFLD pathophysiology [10–12], through ferroptosis reaction on lipids. In turn, it leads to the generation of ROS which is strongly associated with the pathophysiology of the disease. A clinically relevant test would be required to determine whether antioxidant supplementation is effective or not in patients with antioxidant deficiencies. In addition to being inexpensive, voltammetry is a simple, rapid, and portable method and hence is a suitable method for clinical translation [13–15]. The use of voltammetry allows quantitative measurement of the antioxidant activity in fluids and tissues, onsite, and in near-real time [15].

There are various adapted defense mechanisms by the cells such as refuge through antioxidants of low-molecular-weight (LMWAs). [15] The LMWAs either scavenge by neutralizing components of ROS or indirectly by chelation of transition metal. The LMWAs are molecules of smaller size, infiltrate cells frequently, and accumulate in compartments specifically associated with oxidative damage. In human and rat blood, cellular LMWAs are generated by various sources [16].

Plasma is used generally to evaluate tissue damage induced by free radicals. Among the most important targets for evaluating oxidative damage are lipoproteins, which are found in the plasma. Additionally, it contains antioxidants such as uric acid (UA), ascorbic acid (AA), and glutamate like oxidative stress markers, which in total represent a relevant biological milieu [17–20]. Various methods are there to evaluate the antioxidants capacity in the biological system. These methods depend on the capacity of the sample to scavenge a specific ROS [21].

**Figure 1:**
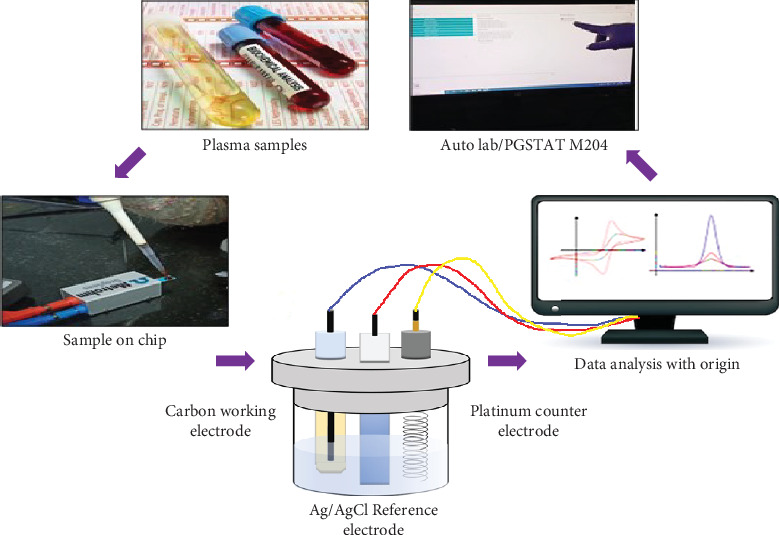
Cyclic voltammetry process.

A component's biological oxidation potential (OP), characterized by its E1/2 value [15]. At a specific OP such as 330 mV for UA and 380 mV for AA, the anodic current can determine the concentration of specific antioxidants [22–24]. Applications of swept potential analytical voltammetry have demonstrated its effectiveness in both research, antioxidant quantification in food such as vanillin [25] and chilli [26] and pharmaceutical [27] quality assessment [28]. Biological samples have been voltametrically analyzed, either *in vivo* or *ex vivo*, for a considerable amount of time by researchers. In tissue homogenate and blood plasma, Chevion et al. developed a CV-based method for quantifying LMWAs as demonstrated in Figure 1. Comparing CV results with high-performance liquid chromatography (HPLC) results, CV results were found to be more reliable [20]. In a study, researchers used a carbon-containing modified electrode (renewable surface) for measuring dietary fat levels in human plasma samples using the same. As a result of CV, tissues and body fluids have been tested for total reducing power (TP) which represents their total antioxidant capacity. This ability plays an important role in preventing tissue damage and inflammation caused by ROS [29, 30].

**Figure 2:**
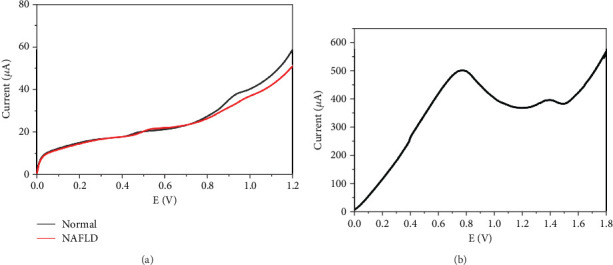
CV tracing of (a) NAFLD versus healthy subject and (b) steatohepatitis patient.

Screen-printed electrodes (SPEs) were tested for AC measurement in the present study. SPEs are disposable microsensors that require a minimal amount of sample volume and are relatively low cost to produce. In addition, such electrodes would allow voltammetry analysis to be portable in some situations [31]. Such examinations were conducted both in normal blood samples and with patient's blood samples (Figure 1). The cyclic calibration curve was obtained from the patient's blood samples due to oxidation followed by AC evaluation. The present study is the direct comprehensive study of NAFLD-mediated oxidative stress on steatosis and steatohepatitis samples with an error of around 1.3% (Figure 2).

## 2. Material and Method

### 2.1. Study Design

This is a retrospective explorative study conducted at Shree Krishna and CHARUSAT hospitals, Anand, India. The newly diagnosed patients with NAFLD and healthy individuals with less than 60 years of age were selected. The patients were showing steatosis and steatohepatitis stage liver disease in USG. Then, 5 mL of fasting patients and healthy individual's blood was collected. At enrolment, out of 107 subjects, 67 cases were diagnosed to be NAFLD cases (patients with fatty liver) and 40 as controls (without NAFLD). The study was initiated as a pilot study including 67 patients. This study involved a range of procedures to collect demographic and medical information from the participants during their visit: A range of questionnaires were introduced during the visit, as well as an anthropometric assessment that was carried out using a stadiometer, a Tabina WB-100A, a Gulick measuring tape, and measurements of height, weight, and waist circumference (best of two readings) done by the help of an anthropologist. A third measurement was done only if the previous two measurements differed by 0.5 cm, 0.1 kg, 0.2 kg, and 2.0 cm, respectively, average of height and weight were used to determine the BMI keeping weight of body in kilogram divided by height in meter square (kg/m^2^) and transverse scan was performed for the purpose of assessing hepatic steatosis as well as hepatic stiffness as a measure of hepatic fibrosis. The parameters taken into consideration are discussed in Table 1.

#### 2.1.1. Human Sample Collection

##### 2.1.1.1. Blood Sample

The peripheral venous blood was collected into two tubes one with ethylene diamine tetraacetic acid (EDTA) anticoagulant to obtain and spin plasma and one with clot tubes for serum. The obtained blood samples were centrifuged for 15 min at 3000 × *g*. Butylated-hydroxytoluene (10 *μ*L/mL) acquired from Sigma-Aldrich, Germany, was added in order to prevent sample oxidation and were stored at −80°C for further use.

##### 2.1.1.2. Tissue Sample Collection

The primary histopathologically confirmed later stage NAFLD patient's 1-cm biopsy specimens from each group of three individuals were selected. The injection fixed wedge biopsy samples utilized during SEM analysis only to confirm the patient's disease stage and used as an example image.

### 2.2. Voltammetric Assessment: Plasma CV Tracing Indicative of Oxidative Stress

CV, an electrochemical tool has been used to analyze the samples. The groups were processed by an Autolab/PGSTAT-M204 standard system with three electrodes. The samples were evaluated using a multielectrode (SPEs) with a thermistor negative temperature coefficient (NTC) purchased from Metrohm dropsens, (Asturias, Spain) of 500-*μ*m ceramic substrate thickness and L33× W10 mm substrate size. In the present work, the anodic polarization curve was recorded using standard SPEs containing circular carbon film as a working electrode, Ag/AgCl film as a reference, and carbon-printed electrode as a counter electrode. The polarization curve was recorded at scan rate of 10 mV/s in the current investigation of blood samples. In the start of the experiment, a cyclic sweep curve of voltammetry has been recorded between 0 and 1.5 V range Ag/AgCl, with the scan rate 10 mV/s. Three replicates were technically performed.

### 2.3. Oxidative Stress Analysis: Nonenzymatic Antioxidants

#### 2.3.1. MDA Estimation

Fresh human serum (250 uL) was mixed with 6 M NaOH (50 uL) and incubated at 60°C for 45 min then 35% perchloric acid (125 *μ*L) was used to acidify. Following hydrolysis, the samples were centrifuged at 15,000 *g* for 10 min. After centrifugation, 250 *μ*L of the supernatant were added to 25 *μ*L of 2, 4-dinitrophenylhydrazine (DNPH) solution. Afterwards, the sample was incubated for 10 min in the dark before being analyzed by Waters Breeze-2 (United States) using ODS2 reverse phase columns. The mobile phase consisted of an acetonitrile and acetic acid-containing HPLC grade water (0.2%). The flow rate 0.5 mL/min and isocratic conditions were maintained. In a UV spectrophotometer at 310 nm, the MDA content in the sample was detected [32].

#### 2.3.2. AGE Determination

Glycation produced synthetic AGE products, and sodium phosphate-buffered saline was used to dilute the serum 500 times. Then, 20 *μ*L viscous serum diluted into 1-mL tube. A Fluoromax spectrometer (Spex instruments) at room temperature was used to record fluorescence spectra, after filtration through Millex-GV filters (0.22 Millipore (*μ*m) pore size). AGE content of plasma was colorimetrically estimated in duplicate at 350/440 nm through AGE-specific fluorescence [33]. Approximately 8.9% of the variance obtained due to interassay variability. The data is expressed in AFU/mg protein.

#### 2.3.3. TOS Analysis

TOS of plasma using triplicate samples was analyzed biochromatically at 560 nm/800 nm depending on the ferrous to ferric ion decomposition reaction in plasma in the presence of oxidants. A fully automatic biochemical analyzer of Hitachi 7600-020 evaluated the ions using xylenol orange as a sample [34]. The data are expressed in *μ*molH_2_O_2_equiv/L.

#### 2.3.4. AOPP Estimation

The extent of oxidative damage to protein products was quantified. Therefore, concentration of AOPP was determined calorimetrically by measuring the sample's capacity to oxidize iodide ions at an absorbance of 340 nm. The remaining portion of samples were then transferred immediately at 4°C and later stored at −80°C for further use.

#### 2.3.5. NO

NO levels, a marker of liver oxidative stress, were determined colorimetrically at 540 nm using an Invitrogen kit (ThermoFisher, United States). Samples remaining were moved to 4°C and then stored at −80°C.

#### 2.3.6. GPx Assessment

The GPx enzyme activity measured by the kit-based assay (make: Invitrogen, United States). The quantitative measurement of peroxidase enzyme was estimated through the anion's absorbance in the solution at 412 nm. The remaining samples were immediately transferred to 4°C and subsequently stored at −80°C for future analysis.

### 2.4. Gene Expression Analysis

Total RNA was extracted from samples using the TRIzol method (Invitrogen, United States), and cDNA was synthesized using the BioradiScript cDNA synthesis kit. Gene expression levels of PNPLA3, PPAR-*α*, TIMP1, Bcl2, IL-6, and TNF-*α* were quantified by qRT-PCR using an Agilent Stratagene Mx3005P machine. SYBR/ROX was used for gene expression detection. RNA concentration and purity were determined using a NanoDrop spectrophotometer (Thermo Fisher Scientific, United States). 18S rRNA served as an endogenous control. Gene expression fold-changes were calculated relative to the control group.

### 2.5. Scanning Electron Microscope

Using OCT compound, tissue sections were carefully cut into 8 × 2 mm sizes and placed on a glass plate without coverslips. An electron microscope was used to analyze the smaller part of the glass slides coated with 2-nm platinum using a sputter coater (JEC-3000FC, Auto Fine Coater, JEOL 6010LA, Japan). SEM images of liver tissue were analyzed using a computerized image analysis system to determine the stage of steatosis.

### 2.6. Data Analysis

#### 2.6.1. NAFLD Characteristics Analysis

The population of study was divided into groups such as early NAFLD/steatosis (patients without fibrosis *n* = 29) and advanced NAFLD/steatohepatitis (patients with steatohepatitis/fibrosis *n* = 38). The steatosis group has normal activity of ALT, while the steatohepatitis group has ≥ 6.6 kPa and upper activity of ALT and abnormal ALT/AST ratio. The clinical characteristics of the population of this study presented in Table 1. The parameters taken into consideration are discussed in the table below.

#### 2.6.2. Dietary Patterns and Nutrition Analysis

All the demographic information collected by the questionnaire. Questionnaires/interviews on diet were of both type qualitative and quantitative. Food frequency questionnaires (FFQ) were used to categorize study respondents' food habits and the frequency of intake. It was validated by the expert committee and followed the National Institute of Nutrition and World Health Organization mentioned guidelines. The daily intake of food was calculated using a 24-h dietary recall. Dietary elements and nutrients with antioxidant properties were given a special consideration in this study. FFQ was used to categorize the study participants based on their diet types (vegetarians: no meat, nonvegetarians: eat meat/fish, and uncategorized: eat meat/fish ≥ 1/month ≤ 1/week) [35–37].

The diet chart comprises 15 items under the major headings as breakfast/dinner cereals, fresh vegetables, fruits, and fruit juices, etc. Four categories of frequency (in points) available for each item: 0 point for 500 g and more, 1 point for 400 g, 2 points for 300 g, and 3 points for 200 g and less, e.g., 1 banana/apple/pear of 150 g approx. and 1 paprika/1 apricot/1 cucumber/1 tomato of 100 g approx. The average food intake of each FFQ food item was calculated by computing the mean of the individual food values for the item, as well as the average amount of energy and macronutrient intake. In this case, the weighted means reflect the frequency with which food items were reported (in g). Based on the 24-h recalls, we calculated the average portion size and nutritional content for food items such as cereals in the FFQ. Thus, the stratum and individual-specific nutrient databases enabled the system to select the appropriate nutrient value for items in FFQ based on participants' groups. A paired *t* test was used to determine statistical significance between the mean estimations from the FFQ and the 24-h dietary recall system.

A linear regression analysis was also performed to calculate energy-adjusted differences between nutrients by using nutrient intake as the dependent variable and the total calories as the adjusted factor. Instead of estimating absolute intakes, FFQs are best used to rank the subjects' intakes. Therefore, the quintile of each participant's intake is determined by comparing the distribution of the entire study population to the standard system and then to the region system, based on the entire study population data. The Cohen's kappa (*κ*) statistic was used to examine quintile agreement between two systems, and the values were interpreted.

#### 2.6.3. Statistical Analysis

Using Nova electrochemical analyzer software 2.0 for the study as well as the Volta metric parameters such as AC for each anodic peak were taken. Antioxidant current is represented as AC_520_, for example indicating the antioxidant oxidation with maximum OP at given AC with ±50 mV. Means and standard deviations (SD) were calculated for continuous variables. Counts and percentages were used to present categorical variables. The chi- square test was used to examine the frequency and statistical significance of qualitative traits across analyzed groups. According to the type of data, paramagnetic or nonparamagnetic tests were performed. For qualitative variables, Mann–Whitney *U* and Kruskal–Wallis ANOVA (one-way) was performed. Statistical analysis was done using GraphPad Prism software 10.4.1 (CA, United States). To analyze the mean value of three groups in the study, ANOVA and post hoc Tukey's multiple comparison test were carried out. The obtained results were shown as odd ratio (OD) and adjusted if required with 95% confidence interval (CI). The Statistical Analysis System (SAS) (9.1, Institute, Inc., Cary, North Carolina) was used to perform statistical analysis. *p* values less than 0.05 were considered significant.

### 2.7. Ethical Consideration

The human samples were collected from Shree Krishna Hospital, Karamsad, with approval number IEC/BU/130/Faculty/12/175/2021 by Institutional Ethics Committee-Bhaikaka University and from CHARUSAT Healthcare and Research Foundation (CHRF), CHARUSAT Hospital, Anand, Gujarat, with approval reference number CHHA/IEC/ADM/21/02/105.27. The test was conducted in accordance with the “National Ethical Guidelines for Biomedical and Health Research involving Human Participants” under section 10 “Human genetic testing and research and ICH Good Clinical Practice E6.” A well-informed consent form was obtained from the participants available in both the languages (English and Gujarati) before collecting the samples.

## 3. Results

### 3.1. Pathophysiological and Dietary Evaluation of Individuals With NAFLD

Factors such as ALT, AST, GGT, and ALT/AST ratio were independently associated with NAFLD condition, whereas in elderly and male patients showed elevated values of gamma-glutamyl transferase (GGT) in case of SHPS only. Participants enrolled for this study were mostly men (56%), with a mean age of 49.3 years, BMI of 31.6 kg/m2, weight of 91.9 kg, and waist circumference of 107 cm. Most patients were employed (71.0%), and were nonvegetarian (73.5%). In terms of vitamins A, E, and C (natural source of antioxidants), vegetarian men attained the highest proportions (79.8%, 63%, and 61%), while unclassified men achieved the lowest proportions (59.9%, 39.5%, and 32.4%, respectively). Overall, these 67 patients' plasma samples were used to check the cyclic voltammograms under NAFLD conditions. Different stages of NAFLD and related profile check for the sample selection have been shown in Table 2.

### 3.2. A Diet History to Evaluate Antioxidant Sources and to Observe Difference in Antioxidant Capacity Between Groups

There was a significant difference between vegetarian and nonvegetarian intake of animal proteins *p* < 0.05 (in both) and cholesterol *p* < 0.01 (in steatosis). According to the dietary regimens of the study subjects, the average daily intake of antioxidant products was presented in Table 3. Comparisons of vegetarians and nonvegetarians showed significant differences. Steatohepatitis patients were consuming less frozen vegetables (*p* < 0.05) compared to others (*p* < 0.001) while potatoes (*p* < 0.05), coffee (*p* < 0.01), and tea (*p* < 0.01) were consumed more by steatosis individuals. Vegetarians consume more fresh vegetables (*p* = 0.001), potatoes (*p* < 0.05), fresh fruit (*p* < 0.01), coffee (*p* < 0.01), and tea (*p* < 0.05) as shown in Table 4.

### 3.3. NAFLD Physiology Voltagrams Representatives Analyzed Through SPEs

Representatives of voltagrams from human samples are shown in Figure 2. Identified anodic peaks for each type of sample with mean SD ± 100 V. The lesser the reductive capacity, the higher the *R* value obtained with significant difference in the peak of participants. The voltammetry curve of the healthy blood (stated as normal) and diseased blood (stated as NAFLD) recorded by SPE at scan rate of 10 mV/s are shown in Figure 2a. The representing voltammetry curves shown two anodic peaks in the blood sample centered near to 0.5 and 0.9 V, demonstrating the working carbon electrode is capable of oxidation of the AA to dehydroasorbic acid, and UA to bis-imine compound. It can be seen that there is significant change in the anodic current for the peak centered at 0.5 V, showing the increase in the concentration of the UA in the progressed disease blood sample. This could be due to the mitochondrial oxidative stress and high fat synthesis in liver cells. The results were recorded for advanced stage sample containing > 400 *μ*mol/L UA and < 20 *μ*mol/L AA detected as shown in Figure 2b. The representing polarization curve demonstrate the similar two oxidation peaks. However, during the process of electrochemical oxidization of UA and AA, the peaks are shifted to higher potential side due to excess concentration of UA, than the actual blood sample with a scan rate of 50 mV/s, which is confirmed by significantly enhanced current values when using fast sweep cyclic voltammetry. Therefore, monitoring UA levels could be a potential marker for steatosis detection.

### 3.4. NAFLD-Induced Severity Analysis of the CV-Generated Data Through Statistical Analysis

**Figure 3:**
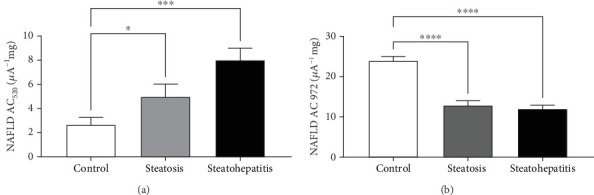
The AC values of control, steatohepatitis plasma samples (HSPS), and steatosis plasma samples (SPS). Data obtained presented here as mean SD represented by ⁣^∗^*p* < 0.05, ⁣^∗∗^*p* < 0.01, ⁣^∗∗∗^*p* < 0.001, and ⁣^∗∗∗∗^*p* < 0.0001.

The corresponding cyclic voltammograms under NAFLD conditions are presented at the scan rate 10 mVs^−1^ in Figure 3 with low peak levels, wherein the healthy individual plasma steady-state anodic currents of voltammetry for oxidative stress were obtained due to convergent transport of mass. No human plasma deficit antioxidants in healthy groups (current: 22.2 *μ*A, 0.48 V), while a severe increase in the plasma AC of 33% in steatohepatitis group's peak for UA (37.8 *μ*A, 0.93) as compared to healthy samples as shown in Figure 3, whereas the second peak indicative of other components such cysteine, carnosine, NAD(P)H, NADH, melatonin, and lipoic acid showed the reduction peak level and current. This depicts the total system antioxidant capacity measurement, where the AC wave comprises several components. CVs of such combinations appear as broader anodic waves along with increase in *la.* The severe spike change in AC_520_ can be seen. This could be due to the changes in the O*P* values of AA as it helps to maintain the homeostasis of circulating and hepatic lipids and provide protection against fatty liver conditions. Low levels of AA indicate high oxidative stress.

### 3.5. NAFLD-Induced Evaluation of Area Covered Through the CV

**Figure 4:**
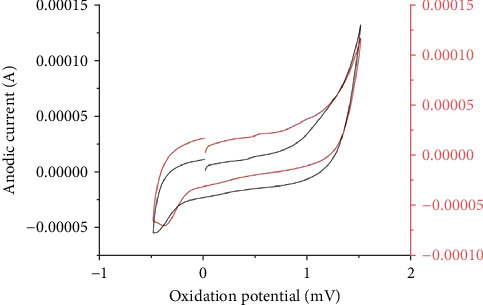
The area covered by the healthy human plasma is demonstrated in black while the area under cover by early NAFLD sample was demonstrated in red.

The evaluated area covered by the *s* was also of varying range. The graph obtained through the diseased subject was comparatively of broader anodic waves and the area under the curve was more as compared to the control. The range of anodic wave of the area under the curve of healthy subjects was 1.08 × 10^−11^ Acm^−2^and 5.4 × 10^−11^ Acm^−2^ of the steatohepatitis sample. Hence, through area under curve (AUC) analysis, researchers may predict the levels of oxidative stress. The higher the oxidative stress, the higher the AUC. This can broaden the peak current. The second anodic peak was affected by the NAFLD presence (*p* = 0.054) as shown in Figure 4. This increases ROS formation and is measured through CV and unravelling the role of the absolute area covered by the cyclic graph obtained. The polarization curve was recorded at scan rate of 10 mV/s in the current investigation of blood samples.

### 3.6. NAFLD-Induced Oxidative Stress Analysis Through Various Methods

#### 3.6.1. NAFLD-Induced Oxidative Stress Analysis via MDA Estimation

**Figure 5:**
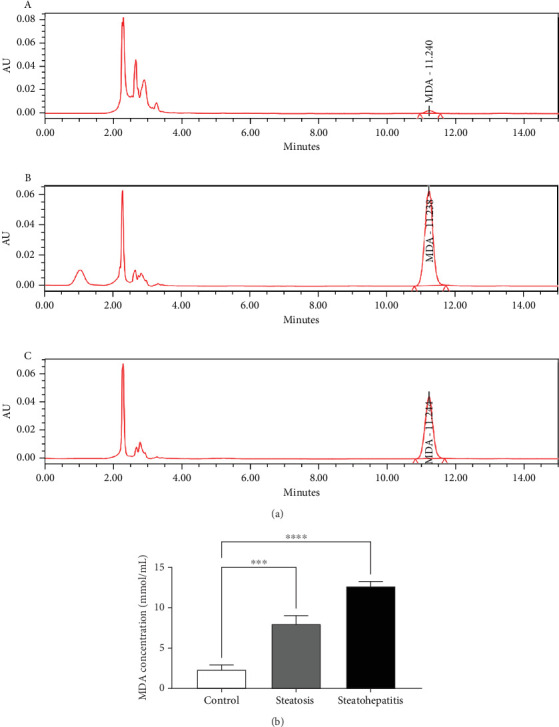
(a) MDA predicting oxidative stress (A) control, (B) steatohepatitis plasma samples (HSPS), and (C) steatosis plasma samples (SPS). Statistical analysis of HSPS and SPS are shown compared to the control group and are demonstrated as ⁣^∗∗^*p* < 0.01, ⁣^∗∗∗^*p* < 0.001, and ⁣^∗∗∗∗^*p* < 0.0001. (b) HPLC chromatograms profile of NAFLD patients showing MDA concentration.

The HPLC chromatograms (Figure 5a,b) of biological samples. MDA levels are represented as follows: (A) control, (B) SHPS, and (C) SPS, were taken within 24 h of sample collection. MDA content was highest in the advanced NAFLD group. The early NAFLD group showed ⁣^∗∗∗^*p* < 0.001 and advanced group ⁣^∗∗∗∗^*p* < 0.0001 w.r.t the healthy group. These results depict the increased free radical generation mediated lipid peroxidation due to NAFLD condition that resulted in MDA overproduction.

#### 3.6.2. NAFLD-Induced Oxidative Stress Analysis Through AGE and TOS Detection

**Figure 6:**
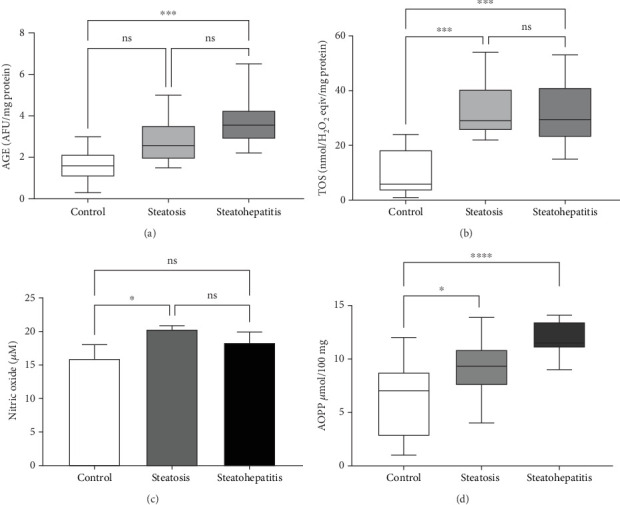
Products of oxidative damage: (a) AGE content, (b) TOS, (c) NO, and (d) AOPP concentration in steatosis plasma samples (SPS) and steatohepatitis plasma samples (SHPS) compared with healthy human subjects. The comparison is done by using Mann–Whitney test. Statistical analysis of SHPS compared to control groups and SPS is demonstrated as ns, ⁣^∗^*p* < 0.05, ⁣^∗∗^*p* < 0.01, ⁣^∗∗∗^*p* < 0.001, and ⁣^∗∗∗∗^*p* < 0.0001.

The NAFLD condition resulted in elevated oxidative stress in circulation. Increased levels of ROS led to lipid fibrinolysis resulting in the production of AGE and TOS. Statistical analysis of plasma levels of AGE (⁣^∗∗^*p* < 0.01) and TOS (⁣^∗∗∗^*p* < 0.001) were significantly distinguished in the steatosis and steatohepatitis stages. The AGE concentrations were 1.56 AFU/mg protein for the control group, 3.79 AFU/mg protein for the early NAFLD group, and 4.07 AFU/mg protein for the advanced NAFLD group. Similarly, in TOS, the obtained concentrations were 29.89 Eqiv/mg protein, 24.6 Eqiv/mg protein, and 8.35 nmol H_2_O_2_ Eqiv/mg protein, respectively (Figure 6).

### 3.7. Evaluation of the Enzymatic Antioxidant Barrier Through GPx Activity

**Figure 7:**
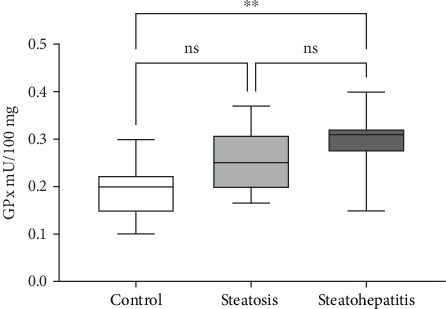
GPx activity comparison between the groups. Data were analyzed using the Mann–Whitney test. Statistical significance compared to the control group is indicated as ns, ⁣^∗^*p* < 0.05, ⁣^∗∗^*p* < 0.01, ⁣^∗∗∗^*p* < 0.001, and ⁣^∗∗∗∗^*p* < 0.0001.

The antioxidant barrier was assessed by measuring the activity of GPx, an enzymatic antioxidant. GPx activity showed a slight increase in tumor tissue relative to normal tissue; however, this difference did not reach statistical significance (Figure 7).

### 3.8. Expression of Inflammation and Lipid Metabolism Genes in NAFLD

The graphs demonstrate that the progression from simple steatosis to steatohepatitis is associated with significant changes in the expression of genes related to fibrosis (TIMP1), lipid metabolism (PNPLA3 and PPAR*γ*), and inflammation (TNF-*α* and IL-6). These findings highlight the key molecular events involved in the pathogenesis of NAFLD and its progression to NASH.

### 3.9. SEM Images of Liver Tissue Distorted Morphology Depicts Steatosis

**Figure 8:**
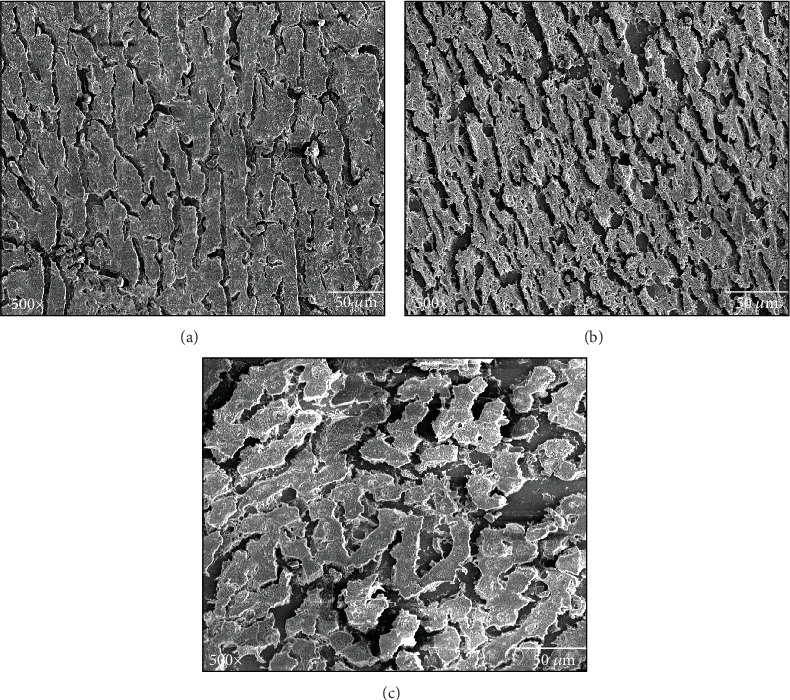
Example image of axial contrast SEM scan slice with the region of interest: (a) control, (b) steatohepatitis plasma samples (SHPS), and (c) steatosis plasma samples (SPS) subjects.

Using scanning electron microscopy, the control group exhibits an intact liver morphology. Conversely, oxidative stress disrupts the hepatocyte cell wall, resulting in apoptosis. Although the cells were intact at 50 microns in the steatosis stage, their sizes were significantly larger than the steatohepatitis stage group (Figure 8).

## 4. Discussion

**Figure 9:**
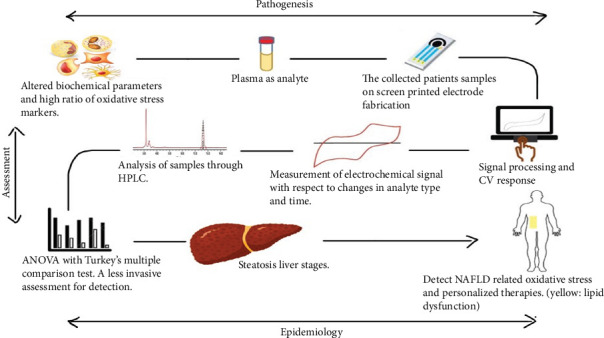
Flow diagram of study sample collection and processing.

Among 67 NAFLD patients, all were included in this study. Based on the severity of their steatosis or the number of complications they had, the NAFLD individuals were categorized into groups listed in Table 2, followed by Tables 3 and 4 describing total energy and type of diet intake by the different groups of patients for better understanding regarding the source of antioxidants. The oxidative stress due to low antioxidant level was measured using CV through plasma (shown in Figure 9). A decrease in their levels were found over time and with increased severity after the NAFLD condition early phase proceeding to advanced stage [3, 32].

Interestingly, each anodic peak represents the existence of potential antioxidants. UA and 2, 6, 8-tri-hydroxy purine comprise the first anodic peak, while carnosine, melatonin, cysteine, glutathione (GSSH/GSSH), NADH, and NADPH [15] are thought to compose the second anodic peak. Hence, ex vivo plasma reflects the antioxidant capacity in vivo before blood is collected, based on the observation earlier explained. The first anodic peak in the human samples was of similar OP to UA suggesting it as a primary component out of many components. The abundance of AA suggests the normal/initial stage and not rapid progression to fibrotic stage as shown in Figure 2a. The patients receiving excessive fat diet (SFA) or in hereditary demonstrate to have increased levels of inflammation, hepatocyte injury, ballooning, and degeneration with or without fibrosis and sometimes the presence of Mallory bodies as shown in Table 3 and Figure 8.

The second AC wave was inflated in NAFLD individuals with early or advanced stage than individuals having normal liver function. Over 60% of patients with steatohepatitis had lower antioxidant levels with soaring complication scores. Following the NAFLD condition, plasma and liver antioxidant levels transiently decreased in studies as recorded in Figure 3. The plasma sample with NAFLD condition deficit maximum antioxidant. Each sample type exhibited two anodic peaks. NAFLD was sensitive to changes in anodic peaks with low Ops as demonstrated in Figure 2a,b. In this case, LMWAs with low OP (for example, ascorbic acid) scavenge more ROS than LMWAs with higher OP (for example, a-tocopherol) [38] and also affects the area under cover as observed in Figure 4.

**Figure 10:**
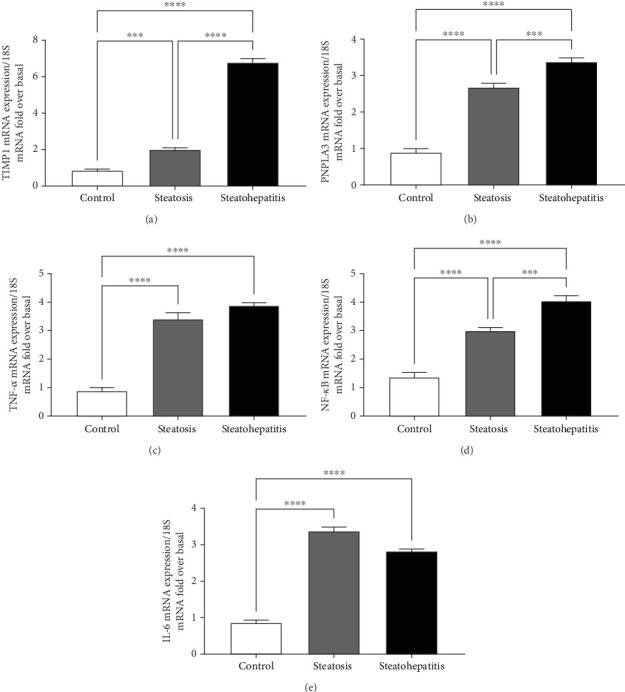
mRNA expression levels of key markers in NAFLD: (a) TIMP1, (b) PNPLA3, (c) TNF-*α*, (d) NF-*κ*B, and (e) IL-6. Statistical significance compared to the control group is indicated as follows: *ns* (not significant), ⁣^∗^*p* < 0.05, ⁣^∗∗^*p* < 0.01, ⁣^∗∗∗^*p* < 0.001, and ⁣^∗∗∗∗^*p* < 0.0001.

NAFLD increases oxidative stress, inflammatory and fibrotic markers (can be electrochemically active) such as TIMP1, PNPLA3, TNF-*α*, NF-*κ*B, and IL-6 in the liver tissue/plasma (as discussed in Figure 10), neurodegeneration, gut disruption, and histological damage in the liver [39], kidney [40], and spleen [41]. As a result, it causes severe systemic responses, which suppresses the immune system and hampers liver recovery such as systemic inflammatory response syndrome (SIRS) later in the disease's course. Consequently, the data depicted in Figure 10 demonstrate and corroborate the hypothesized complex interplay between lipid droplet biogenesis and AC regulation. Researchers can distinguish patients based on their antioxidant deficit using voltammetry, an accessible antioxidant and protein test. Furthermore, this study confirms an earlier finding that individual heterogeneity is widespread at antioxidant levels in plasma under conditions of oxidative stress [42–44]. This would be the first study that involves the South Asian ethnicity from the west coast of India to study variable NAFLD patient plasma samples using CV. In some cases, ambiguous results in antioxidant trials have been attributed to low specificity antioxidant levels in the participants rather than uniformity of treatment. [8, 14] Voltammetry is therefore a viable method for identifying and treating NAFLD patients with redox deficits.

Additionally, the advanced NAFLD scores instigate a decline in second anodic peak and area covered by the leaf formed by the plasma sample. For instance, the recorded CVs of healthy individual and patients' plasma were compared in Figure 4. Elevated oxidative stress leads to lipid peroxidation resulting in MDA formation, an oxidative stress maker. The exacerbated levels of MDA here were measured by HPLC as shown in Figures 5 and 6. The escalating levels of AGE (⁣^∗∗∗^*p* < 0.001), TOS (⁣^∗∗∗^*p* < 0.001), and other markers of lipid peroxidation such as AOPP (⁣^∗∗∗∗^*p* < 0.0001) and NO (⁣^∗∗^*p* < 0.01) strongly suggest increased oxidative stress in NAFLD. This inference is reinforced by the enzymatic evaluation, which showed a significant decrease in GPx activity (⁣^∗∗^*p* < 0.01) as referred in Figure 7 and TIMP1 expression analysis. Given GPx's crucial role in detoxifying oxidative stress by-products, these findings demonstrate compromised enzymatic antioxidant defences in NAFLD.

### 4.1. Recommendations and Limitations

Voltammetry has a few impediments, such as lost effectual changes within the antioxidant profile with time. By selectively degrading enzymes such as uricase and ascorbate oxidase, the components of each anodic wave might have been better identified. The plasma AC varies considerably between the individuals, so a large group size is recommended. A composite baseline could have been formed by obtaining several presurgical samplings and averaging them.

Besides limitations, this study has several acknowledged advantages over other assay techniques for antioxidants that could make it possible to incorporate it into existing NAFLD monitoring methods. In contrast to spectrophotometry-based assays, voltammetry enables monitoring of antioxidant status in near real time. The miniaturization of selective voltammetric electrodes and their placement within small Teflon tubes has shown that continuous or region-specific voltammetric data is possible. The SPE is a remarkable step towards therapeutic application of this technology. Unlike classic voltammetry methods, SPEs do not require the time-consuming defouling phase. Clinical and hazardous biological sample contaminants can be reduced by using disposable sensors. The present study requires approx. Then, the 10-*μ*L sample volume as compared to traditional methods (glassy carbon electrode) where the volume of the required sample is approx. Then, 250 *μ*L–1 mL [15]. Additionally, SPE can be assessed using portable potentiostats.

## 5. Conclusion

In the initial stages of NAFLD, the first anodic peak in cyclic voltammetry exhibits a moderate change, likely reflecting fluctuations in the concentration of readily oxidizable antioxidants. Conversely, the second anodic peak shows a progressive decline, suggesting a reduction in the availability of molecules such as thiols, catecholamines, flavanols, and transition metals. This pattern reverses in the later stages of the disease, particularly in steatohepatitis. The first anodic peak significantly increases, correlating with elevated levels of purine metabolism by-products, notably UA, which acts as a pro-oxidant. This shift aligns with the established pro-oxidant environment characteristic of advanced NAFLD/NASH. Therefore, clinicians may be able to distinguish between steatosis and steatohepatitis condition using plasma AC_520_ and AC_972_. Additionally, the SPE approach provides an alternate method for assessing antioxidant status and directing antioxidant therapy and dietary guidelines in other NAFLD-related and systemic disorders.

## Figures and Tables

**Table 1:** Flow table of participant recruitment criteria.

**Sample**	**Blood sample: total of 67 patients screened with fatty liver condition with regular blood investigation an USG. 5-mL fasting blood was collected to avoid extra prick to the patients from veins in EDTA and SST vacutainer by the expert phlebotomist (testing was done after appropriate pretest counselling and if they were willing to give informed consent)**	
	**NAFLD**	
**Healthy control**	**Steatosis**	**Steatohepatitis**
*Inclusion*		
Clinical parameters Individuals of 18–60 years with no liver damage, normal levels of AST, ALT, normal levels of bilirubin and albumin. USG parameters Absence of fatty liver.	Clinical parameters 1) 18–60 years obese/nonobese with 20–40 g/day or less alcohol consumption for 10–05 years (nonobese, BMI < 25; obese, BMI ≥ 25) 2) Steatosis diagnosed/USG 3) ↑ ALT/AST (−/+) 4) ↑ bilirubin (−/+) USG parameters Fatty liver is present	Clinical parameters 1) 18–60 years obese/non obese with 20–40 g/day or more alcohol consumption for more than 10–05 years (nonobese, BMI < 25; obese, BMI ≥ 25) 2) Small liver in USG 3) ↑ ALT/AST (−/+) 4) ↑ bilirubin (−/+) 5) ↓ serum albumin USG parameters Steatohepatitis is present

*Exclusion*		
ALT/AST ratio altered Liver or other diseases On any drug/specific regimen or antioxidant supplements Pregnant or breastfeeding women On a specific regimen or took antioxidant supplements such as vitamin C, E, coenzyme Q10, N-acetyl cysteine and/or selenium	60 g/day or more alcohol consumption Drug-induced hepatitis or liver Pregnant or breastfeeding women Positive hepatitis virus markers or autoantibodies	

*Note:* Role of physician: to diagnose the condition of individual (control or patient), sample collection, and hand over. All the samples were stored at 4°C and −20°C for further investigation. Diagnosis baseline by ultrasound. Fatty liver present: mild to a significant increase in the liver echogenicity with poor or no echogenicity of the portal vein wall or right lobe of liver, > 1 AST/ALT ratio.

*Abbreviations:* BMI: body mass index, EDTA: ethylene diamine tetraacetic acid, g: gram.

**Table 2:** Clinical characteristics of NAFLD Patients.

**Parameters**	**Control (40)**	**Steatosis (29)**	**Steatohepatitis (38)**
Gender (M/F)	23/17	19/9	19/20
Age (years)	39 (30–49)	49 (38–60)	56 (47–60)
BMI	22.8 (19.0–24.9)	30.2 (27.7–34.1)	31.0 (27.9–35.0)
WHR	0.84 (0.8–0.88)	0.90 (0.85–0.94)	0.94 (0.91–0.99)
AST (IU/L)	21 (15–25)	30 (22–36)	47 (33–72)
ALT (IU/L)	27 (17–33)	32 (24–38)	75 (58–121)
GGT (IU/L)	20 (0–30)	36 (21–62)	62 (32–116)
Diet (%)			
Vegetarian (%)	26 (65.5)	5 (2.4)	13 (17.8)
Nonvegetarian (%)	9 (23.3)	21 (73.5)	24 (6.7)
Unclassified (%)	3.9 (9.7)	3 (9.2)	1 (1.5)

*Abrreviations:* WHR: waist–hip ratio; GGT: gamma-glutamyl transferase.

**Table 3:** Energy, macronutrients, cholesterol, and fiber intake per day by the selected subjects.

**Parameters**	**Control (40)**	**Steatosis (29)**	**Steatohepatitis (38)**
Fiber, g	27.3 ± 10.1	16.9 ± 8.2^∗^	15.3 ± 7.6^∗^
Cholesterol, mg	201.0 ± 155.4	235.9 ± 198.4^∗^	350.2 ± 231.1^∗^
*α*- Linoleic acid	1.3 ± 1.9	1.6 ± 1.45	2.1 ± 1.7^∗^
Linoleic acid	8.6 ± 6.1	10.1 ± 5.5^∗^	12.4 ± 7.5^∗^
Fat (%)	27.5	33.9⁣^∗^	38.1⁣^∗^
SFA, g	30.4 ± 21.6	34.9 ± 34.6^∗^	38.1 ± 20.6^∗^
Total fat, g	69.4 ± 34.2	82.63 ± 44.2	105.7 ± 51.4^∗^
Protein (%)	14.7	12.0	11.3⁣^∗^
Plant protein	39.8 ± 19.0	21.3 ± 8.8^∗^	20.5 ± 9.6^∗^
Animal protein	15.9 ± 10.5	41.2 ± 21.9^∗^	57.5 ± 29.8^∗^
Carbohydrate, g	271.1 ± 125.0	283.7 ± 113.8	312.54 ± 124.6^∗^
Energy, Kcal	2005.6 ± 975	2011.54 ± 993.0^∗^	2543.1 ± 879.8^∗^

*Abbreviation:* SFA: saturated fatty acid.

Post hoc analysis: ⁣^∗^significant difference between stages as compared to the control group.

**Table 4:** Antioxidants containing food products' intake as a natural source by the selected subjects.

**Parameters**	**Control (40)**	**Steatosis (29)**	**Steatohepatitis (38)**
Breakfast cereals, g	8.7 ± 28.9	3.59 ± 14.9	2.9 ± 17.9^∗^
Dinner cereals, g	5.9 ± 47.9	4.3 ± 59.8	11.2 ± 71.2^∗^
Coffee, mL	111.9 ± 183.7	149.9 ± 183.4^a∗^	187.6 ± 189.7^a∗^
Dried fruits, g	0.5 ± 7.9	0.47 ± 6.3	0.00
Egg, g	5.3 ± 52.4	4.3 ± 43.1	3.2 ± 41.1^∗^
Fresh vegetables, g	349.7 ± 346.81	151 ± 156.9^∗^	133.7 ± 94.3^∗^
Fresh fruits, g	233 ± 279.0	198 ± 223.54	145 ± 212.35^∗^
Groats, g	9.70 ± 33.7	6.85 ± 20.5	5.72 ± 14.7^∗^
Juice, mL	47.1 ± 133.0	41.8 ± 126.5	37.5 ± 152.4
Legumes, g	10.7 ± 19.2	4.8 ± 39.3	3.2 ± 15.9^∗^
Nuts, g	6.71 ± 12.1	1.29 ± 7.8	0.99 ± 67
Potatoes, g	143.±213.7	271.6 ± 269.3^∗^	383.9 ± 208.0^∗^
Processed vegetables, g	37.1 ± 71.1	33.4 ± 65.1^∗^	20.3 ± 39.1^∗^
Seeds, g	0.58.3 ± 4.3	0.44 ± 5.6	0.00
Milk/tea, mL	336.9 ± 351.6	301.9 ± 227.3^∗^	247.96 ± 253.5^∗^
Whole meal bread, g	27.6 ± 59.00	21.58 ± 64.1	18.00 ± 41.4

Post hoc analysis: ⁣^∗^significant difference between stages as compared to control group.

## Data Availability

The data that support the findings of this study are available from the corresponding author upon reasonable request.
